# TLC599 in patients with osteoarthritis of the knee: a phase IIa, randomized, placebo-controlled, dose-finding study

**DOI:** 10.1186/s13075-022-02739-4

**Published:** 2022-02-21

**Authors:** David J. Hunter, Chi-Ching Chang, James Cheng-Chung Wei, Hsiao-Yi Lin, Carl Brown, Tien-Tzu Tai, Chih-Feng Wu, Wing Chia-Ming Chuang, Sheue-Fang Shih

**Affiliations:** 1 Institute of Bone and Joint Research, Kolling Institute, University of Sydney, Sydney, Australia; 2 Rheumatology Department, Royal North Shore Hospital, St Leonards, NSW 2065 Australia; 3 Division of Allergy, Immunology and Rheumatology, Department of Internal Medicine, School of Medicine, College of Medicine, Taipei Medical University, Taipei City, Taiwan; 4 Division of Rheumatology, Immunology and Allergy, Department of Internal Medicine, Taipei Medical University Hospital, Taipei City, Taiwan; 5 Institute of Medicine, Chung Shan Medical University, Taichung City, Taiwan; 6 Division of Allergy, Immunology and Rheumatology, Chung Shan Medical University Hospital, Taichung City, Taiwan; 7 Graduate Institute of Integrated Medicine, China Medical University, Taichung, Taiwan; 8 Department of Medicine, Cheng Hsin General Hospital, Taipei City, Taiwan; 9 School of Medicine, National Yang-Ming University, Taipei City, Taiwan; 10 Taiwan Liposome Company, Ltd., 2F, No. 3 Yuanqu St., Nangang Dist., Taipei City, 115 Taiwan

**Keywords:** Knee osteoarthritis, Glucocorticoid, Pain, WOMAC, VAS

## Abstract

**Background:**

Corticosteroid injection for knee osteoarthritis is limited by its modest duration of treatment effect. The liposome formulation of dexamethasone sodium phosphate (TLC599) was developed for the sustained relief of osteoarthritis pain. This clinical study was conducted to evaluate the efficacy and safety of TLC599 at two dose levels in patients with knee osteoarthritis.

**Methods:**

A randomized, double-blinded, placebo-controlled study was conducted in 75 patients with osteoarthritis of the knee from 13 study centers. Patients were randomized and administered a single intra-articular injection of TLC599 or placebo and assessed for efficacy and safety for 24 weeks. Patient-reported outcomes included the Western Ontario and McMaster Universities Arthritis (WOMAC) Index for pain and function and visual analog scale for pain.

**Results:**

TLC599 at 12 mg demonstrated significantly greater reduction in WOMAC pain through 12 weeks (least squares (LS) mean difference = − 0.37, *p* = 0.0027) and through 24 weeks (LS mean difference = − 0.35, *p* = 0.0037) when compared to placebo. TLC599 12 mg also exhibited significantly greater improvement in function when compared to placebo at 24 weeks (LS mean difference = − 0.26, *p* = 0.0457). TLC599 18 mg did not significantly improve pain or function in comparison with placebo. The use of acetaminophen during the study was less in both TLC599 groups in comparison with placebo. No major or unexpected safety issues were reported.

**Conclusions:**

In participants with symptomatic knee osteoarthritis, TLC599 is a well-tolerated treatment that reduces pain and improves function for up to 24 weeks, a longer duration than that reported for existing IA treatments.

**Trial registration:**

ClinicalTrials.gov, NCT03005873. Registered on 29 December 2016

## Background

Osteoarthritis (OA) is a rheumatic musculoskeletal disorder with a high prevalence and affects more than 200 million people worldwide [1]. As the most common form of arthritis, OA is the leading cause of chronic disability and reduced activity in elders [2]. The Osteoarthritis Research Society International (OARSI) also describes OA as a serious disease [3]. OA affects the synovial joints including those of the knee, hands, hip, and spine, and patients with knee OA accounts for a large proportion of OA cases. In particular, knee OA is characterized by articular and subchondral bone cartilage degradation and osteophyte formation, leading to joint pain, impairment in movement, and reduction in physical function or daily activities [4]. It was estimated that 14 million people in the US have symptomatic OA [5], and around 7.7 million have advanced radiographic knee OA, as characterized by a Kellgren-Lawrence (KL) grading score of 3–4; almost half are between 45 and 64 years of age.

Treatments for knee OA primarily involve a combination of exercise and lifestyle modification, pharmacological treatment, over-the-counter supplements, and surgical joint replacement. Non-surgical treatments are usually effective for patients in the early stages of OA (KL grades 1 to 3), while surgical treatment with joint replacement is frequently a choice for patients with end-stage knee OA. Pharmacological treatments, such as acetaminophen, non-steroidal anti-inflammatory drugs (NSAIDs), cyclooxygenase-2 inhibitors, corticosteroid injections, and tramadol are used by OA patients for symptom relief [6, 7]. In particular, there is an increasing trend in opioid prescription to treat OA pain, with poor patient satisfaction, and increased morbidity and mortality [8]. Intraarticular (IA) corticosteroids are recommended as a standard treatment for OA of the knee, though treatment effect is of modest duration, often for only 2 to 4 weeks [9]. Furthermore, repeat injections are normally limited to 4 injections annually [10, 11], and it was shown that repeated quarterly IA corticosteroids in knee OA for 2 years were associated with more cartilage loss than saline injection [12]. A non-opioid treatment that could effectively reduce pain and provide sustained pain relief could fulfill a large unmet need.

TLC599 is a liposome formulation of dexamethasone sodium phosphate (DSP), a water-soluble and potent glucocorticoid. It was developed with the BioSeizer platform technology [13] to prolong the local residence of DSP in the joint space, with the potential to reduce injection frequency and risk, while maximizing clinical benefit. The objective of the current clinical study was to evaluate the efficacy and safety of a single IA injection of TLC599 over a period of 24 weeks in participants with OA pain of the knee.

## Methods

The aim of this study was to evaluate the treatment efficacy of different test doses of TLC599 in patients with symptomatic knee OA. The study was a randomized, double-blinded, placebo-controlled phase IIa clinical trial (ClinicalTrials.gov no.: NCT03005873) to test two TLC599 doses (12 mg and 18 mg DSP) compared to placebo in participants with knee OA. The study protocol, all study protocol amendments, investigator’s brochure, informed consent form, and any other relevant documents were reviewed and approved by an independent ethics committee (IEC) or institutional review board (IRB) at each study center. The study was conducted in accordance with the approved protocol, the ethical principles derived from international guidelines including the Declaration of Helsinki (2013), International Council for Harmonisation, Good Clinical Practice Guidelines, and applicable laws and regulations. An informed consent form was signed by the participant or representative before they entered the study. Thirteen study centers (5 in Australia and 8 in Taiwan) participated in the study, and approximately 72 participants were planned for enrollment. The study was conducted between May 2017 and July 2018.

### Eligibility

Key inclusion criteria for eligible subjects included males or females ≥ 50 years of age with OA associated symptoms for ≥ 6 months, OA confirmation based on the American College of Rheumatology Criteria for Classification of Idiopathic OA of the knee, a radiographic KL grade 2 or 3 for knee OA severity, and a visual analog scale (VAS) self-reported pain score between 5.0 and 9.0 (out of 10) in the study knee. Key exclusion criteria included the use of systemic corticosteroids within 30 days prior to dosing; glucosamine, chondroitin, or dietary supplement with unstable dose or frequency within 4 weeks before screening; IA corticosteroid, hyaluronic acid, and other IA injection within 3 months prior to screening; chemotherapeutic or systemic immunosuppressant agents for inflammatory diseases; investigational agents within 6 months prior to screening; and concurrent use of anticoagulants. Participants with any use of new rehabilitation or exercise program within the specified time frame before or during screening were also excluded. Also, the use of prohibited medications other than acetaminophen and oral NSAIDS within 48 h and 7 days, respectively, prior to dosing was excluded. Other exclusion criteria associated with participant’s health condition included autoimmune diseases, IA bleeding, infective arthritis or gout attack, amputation of the lower limb, unstable knee joint, acute injury to the study knee in the prior 6 months, any surgery or arthroscopy in the study knee in the prior 12 months, acute infection or infection-related inflammation (non-study knee), skin lesion or breakdown at the injection site, body mass index > 40 kg/m^2^, low platelet count or blood coagulation disorder, history of acquired or congenital immunodeficiency diseases, concurrent or uncontrolled infectious disease, history of treated malignancy with disease free for ≤ 5 years, stroke or myocardial infarction, uncontrolled and unstable concurrent medical or psychiatric illness, allergy or hypersensitivity to the study drug, pregnancy, and pre-defined laboratory abnormalities.

### Study treatment, randomization, and concealment

For each participant, one knee was selected and defined as the study knee to receive study treatment. The study knee was determined based on the presentation of symptoms associated with OA for 6 months prior to screening visit with an equal or higher VAS pain score than the non-study knee. If the VAS score for both knees diagnosed as OA was equal, the study knee was selected by the investigator with reason documented. Eligible participants were randomized in a ratio of 1:1:1 (block size = 6) using an interactive web response system (IWRS, Cenduit Interactive Response Technology), with stratification based on bilateral (VAS pain score ≥ 3 in the non-study knee) or unilateral (VAS pain score < 3 in the non-study knee) knee pain, to receive a single IA injection of one of the following three blinded treatments at baseline: (1) TLC599 at 12 mg DSP with 100 μmol phospholipid (PL) (TLC599 12 mg; 1.0 mL), (2) TLC599 at 18 mg DSP with 150 μmol PL (TLC599 18 mg; 1.5 mL), or (3) placebo (1.5 mL normal saline) at day 0.

The study included blinded and unblinded teams; only the unblinded team responsible for study drug injection had access to the study drug identity. The study treatments were stored in boxes attached with tamper-evident tape while the dosing syringe was wrapped with non-transparent tape to ensure the blindness of the participants. The unblinded injector performed the study drug injection using a 21-gauge needle under aseptic conditions.

Following injection of the study medication, only acetaminophen up to 3 g/day was permitted as pain rescue medication during the study but was not to be used within 48 h prior to efficacy assessment at scheduled visits. Other pain medications including NSAIDs and opioids were not permitted.

### Outcome measures and follow-up visits

Efficacy assessments were based on measurement outcomes at the scheduled study visits using patient-reported questionnaires, including the Western Ontario and McMaster Universities Arthritis Index (WOMAC)-Pain and WOMAC-Function subscales, and patient-reported pain on a VAS. The WOMAC is a self-administered 24-item scale (each on a Likert scale of 0 to 4, with higher scores indicating worse symptoms, divided into subscales of pain, stiffness, and function; average score in each subscale was calculated for analysis). Eligible subjects were scheduled for study visits on day 0 (baseline), day 3, and weeks 1, 4, 8, 12, 16, 20, and 24. The outcome measures with participant-administered questionnaires were conducted at each study visit.

The primary endpoint was the change from baseline in WOMAC-Pain score through week 12. Secondary analyses included change from baseline in WOMAC-Pain, WOMAC-Function, and VAS pain scores at various time points up to week 24, as well as the proportion of clinically durable responders (defined as > 30% pain reduction as measured by the WOMAC-Pain scale in every subsequent post-dosing visit). The EuroQol-5 Dimension (EQ-5D) for assessing the quality of life was also completed by the participants at the scheduled visits. Daily acetaminophen consumption (prohibited within 24 h prior to the study visits) was reported by the participants using a paper diary.

Safety assessment of the study drugs was based on the collection of adverse events, physical examination, clinical chemistry and hematology, urinalysis, 12-lead electrocardiogram (ECG), hemoglobin A1c (HbA1c), blood cortisol, and magnetic resonance imaging (MRI) of both knees.

### Sample size

No formal sample size calculation was performed for this study, as this was the first randomized, blinded, and placebo-controlled study conducted with the study drug TLC599. It was anticipated approximately 20 participants in each study group would complete 24 weeks of follow-up after study drug dosing. A drop-out rate of 15% was expected, requiring approximately 72 subjects enrolled in the study.

### Statistical analyses

Efficacy analyses were performed using the modified intent-to-treat population (mITT) data, which included all randomized participants who received a complete dose of TLC599 or placebo and had at least 1 complete efficacy evaluation after study drug dosing. The changes from baseline in WOMAC-Pain, WOMAC-Function, and VAS pain scores were analyzed using a mixed-effects model for repeated measures (MMRM) with restricted maximum likelihood estimation. The model included factors of treatment, visit (as a categorical variable), and baseline value as fixed factors; center as a random factor; and treatment-by-visit as interaction terms. Changes from baseline at each time point, and for intervals through defined time points, as well as differences between treatment groups and placebo, were estimated using this methodology. Clinically durable responders were analyzed using a logistic regression model including treatment group and baseline value of WOMAC-Pain. The least squares means and 2-sided 90% confidence intervals were reported, and all statistical assessments were conducted 1-sided and evaluated at the 5% level of significance; analyses were performed using SAS version 9.4.

The safety data were summarized for all participants who received any dose of the study drug (safety population). No interim analysis was planned in this study, and all analyses were conducted after the database lock per statistical analysis plan.

For primary and secondary efficacy analyses, missing data was not imputed. All data collected was included in the assessment of patient safety, while missing or incomplete AE data assumed the greatest relationship to study drug and/or severity.

## Results

### Demographic characteristics

**Fig. 1:**
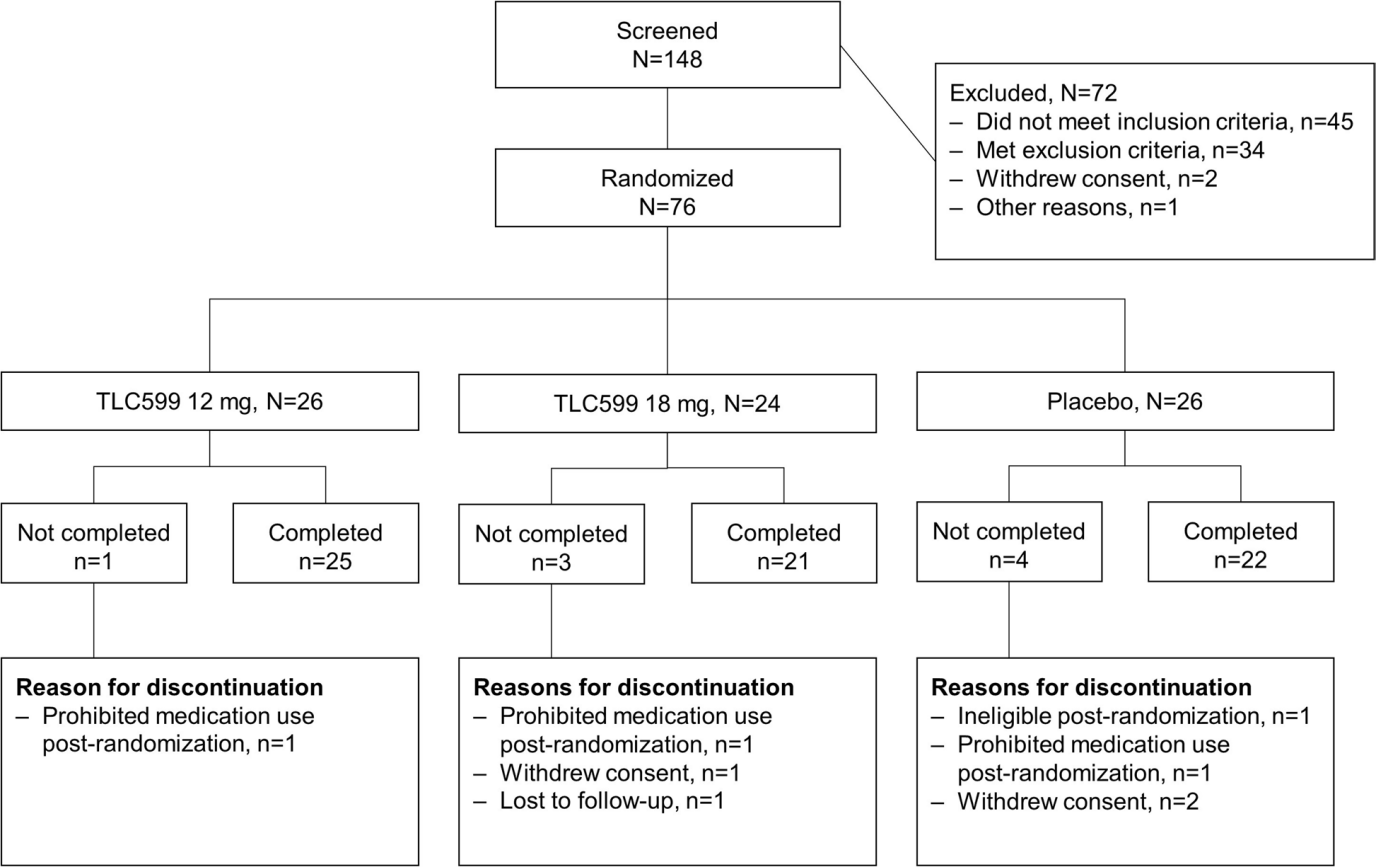
Participant disposition

A total of 148 participants were screened for participation and there were 72 screen failures. The 76 participants in the intent-to-treat population (all randomized patients) included 26 participants assigned to placebo, 26 to TLC599 12 mg, and 24 to TLC599 18 mg (Fig. 1). One participant assigned to placebo was found to be ineligible after randomization, was withdrawn prior to receiving study treatment, and was not included in the safety and mITT populations.

In the safety population (*n* = 75), the majority (60.0%) of participants were aged 50 to 65 years, and the mean age was 63.9 years. There were more female (66.7%) than male (33.3%) participants. Study participants were mostly Asian (from Taiwan, 50.0%) or Caucasian (from Australia, 50.0%). Demographic characteristics were generally balanced among the treatment groups, although there were somewhat greater proportions of males and participants with KL scores of grade 2 in the TLC599 12 mg group (Table 1).

**Table 1:** Baseline demographic and clinical characteristics (safety population)

Characteristic	Statistics	Placebo (***n*** = 25)	TLC599 12 mg (***n*** = 26)	TLC599 18 mg (***n*** = 24)
**Age** (years)	Mean (SD)	64.8 (8.45)	63.9 (9.07)	62.9 (8.80)
**Gender**				
Male	*n* (%)	7 (28.0)	11 (42.3)	7 (29.2)
Female	*n* (%)	18 (72.0)	15 (57.7)	17 (70.8)
**Race**				
Asian	*n* (%)	12 (48.0)	13 (50.0)	12 (50.0)
Australian aboriginal	*n* (%)	0	0	1 (4.2)
Caucasian	*n* (%)	13 (52.0)	13 (50.0)	11 (45.8)
**Country**				
AU	*n* (%)	13 (52.0)	13 (50.0)	12 (50.0)
TW	*n* (%)	12 (48.0)	13 (50.0)	12 (50.0)
**BMI** (kg/m^2^)	Mean (SD)	27.93 (4.655)	27.65 (4.286)	27.96 (5.114)
**Baseline VAS score** (study knee)	Mean (SD)	6.56 (1.049)	6.45 (1.113)	6.87 (1.215)
**Baseline WOMAC-Pain subscale score** (0–4)	Mean (SD)	1.62 (0.609)	1.49 (0.558)	1.74 (0.631)
**Baseline WOMAC-Function subscale score** (0–4)	Mean (SD)	1.48 (0.652)	1.53 (0.531)	1.82 (0.742)
**Knee pain**				
Bilateral	*n* (%)	15 (60.0)	16 (61.5)	15 (62.5)
Unilateral	*n* (%)	10 (40.0)	10 (38.5)	9 (37.5)
**KL grade**				
2	*n* (%)	9 (36.0)	13 (50.0)	9 (37.5)
3	*n* (%)	16 (64.0)	13 (50.0)	15 (62.5)

*AU* Australia, *BMI* body mass index, *n* number of participants, *SD* standard deviation, *TW* Taiwan

### Efficacy

**Fig. 2:**
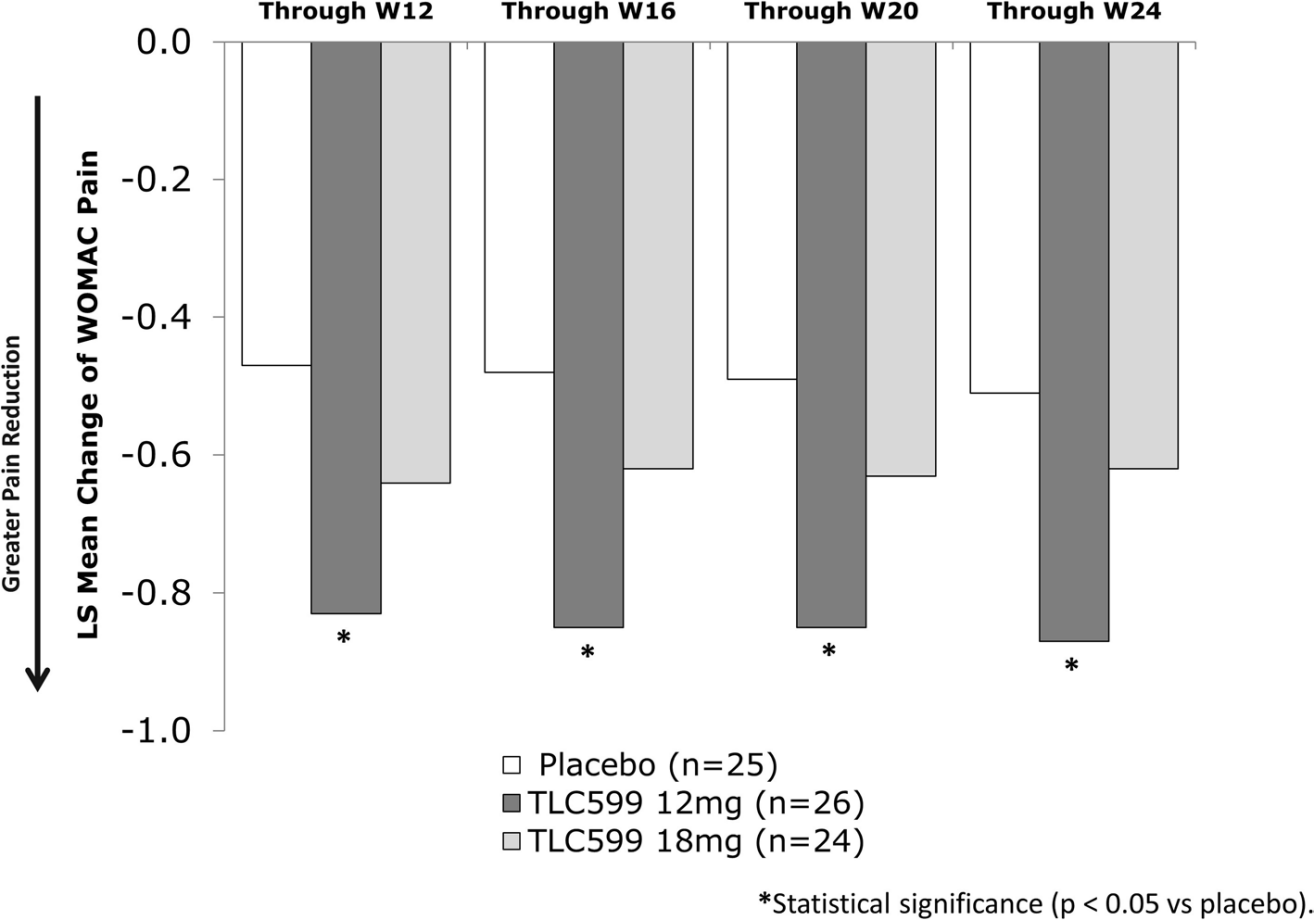
The mean change from baseline in WOMAC-Pain scores through scheduled visits. *p < 0.05. LS, least squares

Based on the mITT population (*n* = 75), participants receiving a single IA dose of TLC599 12 mg demonstrated a statistically significant improvement in WOMAC-Pain score compared to placebo through week 12 (*p* = 0.0027), meeting the study’s primary endpoint (Fig. 2; Table 2). Further, the improvement was persistent, with statistical superiority compared to placebo, reflecting a sustained duration of pain control through week 24. Improvements in WOMAC-Pain with TLC599 18 mg did not reach statistical significance compared with placebo for any time interval.

**Table 2:** Statistical analysis on the mean change from baseline in WOMAC-Pain scores through scheduled visits

Interval	Treatment	Number	LS mean (SE)	90% CI	Difference vs placebo		
					LS mean (SE)	90% CI	***p***-value
D3 - W12	Placebo (*N* = 25)	25	− 0.47 (0.092)	(− 0.619, − 0.313)			
	TLC599 12 mg (*N* = 26)	26	− 0.83 (0.090)	(− 0.984, − 0.685)	− 0.37 (0.129)	(− 0.583, − 0.154)	0.0027
	TLC599 18 mg (*N* = 24)	24	− 0.64 (0.094)	(− 0.794, − 0.481)	− 0.17 (0.131)	(− 0.390, 0.047)	0.0971
D3 - W16	Placebo (*N* =2 5)	25	− 0.48 (0.091)	(− 0.633, − 0.332)			
	TLC599 12 mg (*N* = 26)	26	− 0.85 (0.088)	(− 0.999, − 0.705)	− 0.37 (0.127)	(− 0.580, − 0.158)	0.0024
	TLC599 18 mg (*N* = 24)	24	− 0.62 (0.093)	(− 0.772, − 0.463)	− 0.13 (0.129)	(− 0.350, 0.080)	0.1498
D3 - W20	Placebo (*N* = 25)	25	− 0.49 (0.091)	(− 0.647, − 0.343)			
	TLC599 12 mg (*N* = 26)	26	− 0.85 (0.089)	(− 1.000, − 0.704)	− 0.36 (0.127)	(− 0.570, − 0.145)	0.0033
	TLC599 18 mg (*N* = 24)	24	− 0.63 (0.093)	(− 0.782, − 0.472)	− 0.13 (0.130)	(− 0.349, 0.084)	0.1558
D3 - W24	Placebo (*N* = 25)	25	− 0.51 (0.092)	(− 0.666, − 0.361)			
	TLC599 12 mg (*N* = 26)	26	− 0.87 (0.089)	(− 1.014, − 0.718)	− 0.35 (0.128)	(− 0.565, − 0.139)	0.0037
	TLC599 18 mg (*N* = 24)	24	− 0.62 (0.093)	(− 0.780, − 0.469)	− 0.11 (0.130)	(− 0.328, 0.106)	0.1985

LS means, CIs, and *p*-values were obtained from an MMRM model including factors of treatment, visit, and baseline value as fixed factors, center as a random factor, and treatment by visit as interaction terms. *CI* confidence interval, *D* day, *LS* least squares, *mITT* modified intent to treat, *MMRM* mixed effect model repeat measurement, *SE* standard error, *W* week

**Fig. 3:**
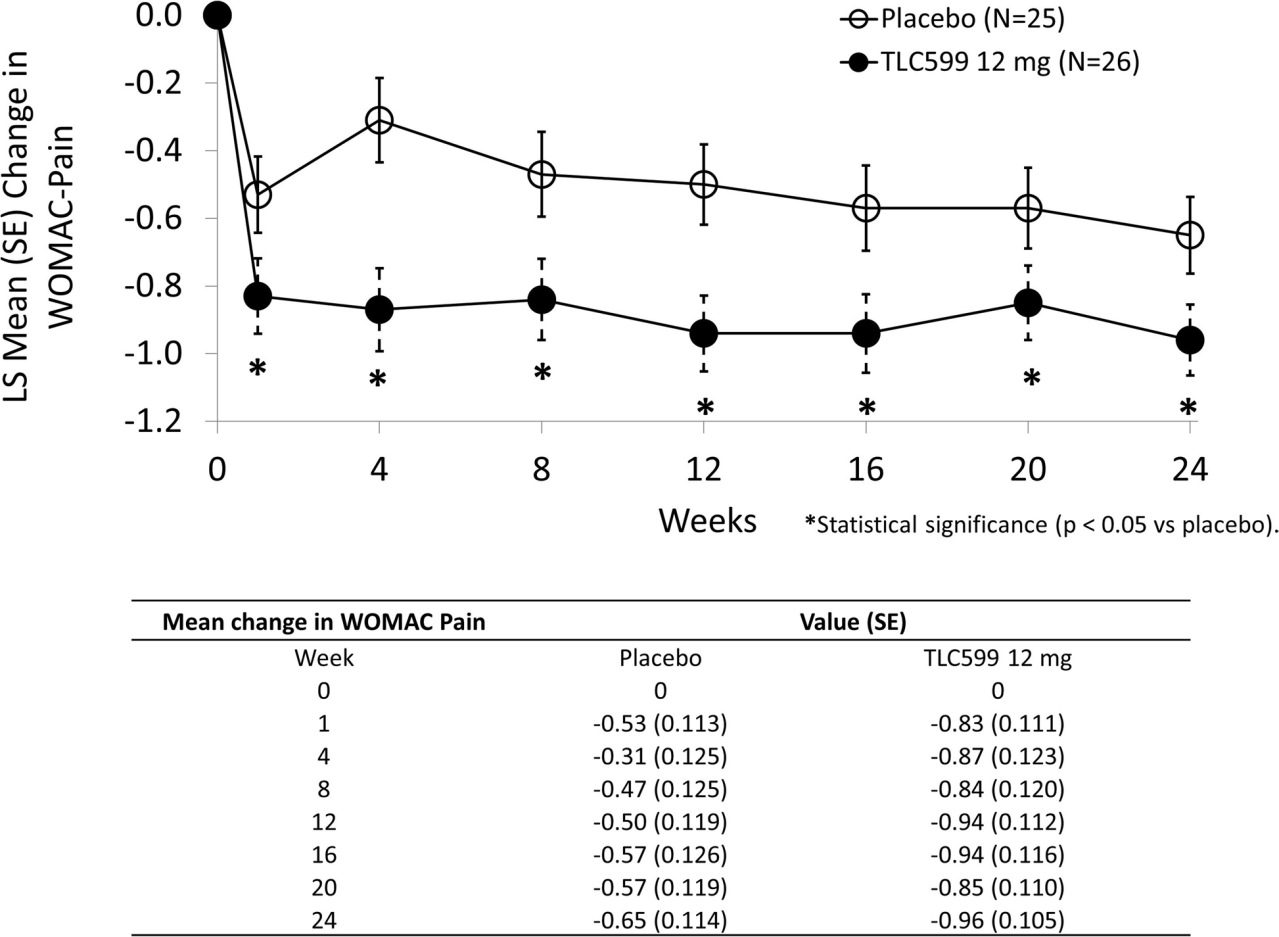
The mean change from baseline in WOMAC-Pain scores at scheduled visits. *p < 0.05. LS, least squares; SE, standard error

Using a landmark analysis at each assessment visit against baseline, participants receiving TLC599 12 mg demonstrated a statistically significantly greater reduction in pain compared to placebo at all post-dosing visits (Fig. 3); those receiving TLC599 18 mg only achieved a significant reduction in pain versus placebo at week 4.

The percentage of durable responders was significantly greater in the TLC599 12 mg group (56.0%) compared with the placebo group through week 12 (56.0% vs 28.6%; *p* = 0.0100) and through week 24 (52.0% vs 22.2%; *p* = 0.0143); differences between TLC599 18 mg and placebo groups did not reach statistical significance.

**Fig. 4:**
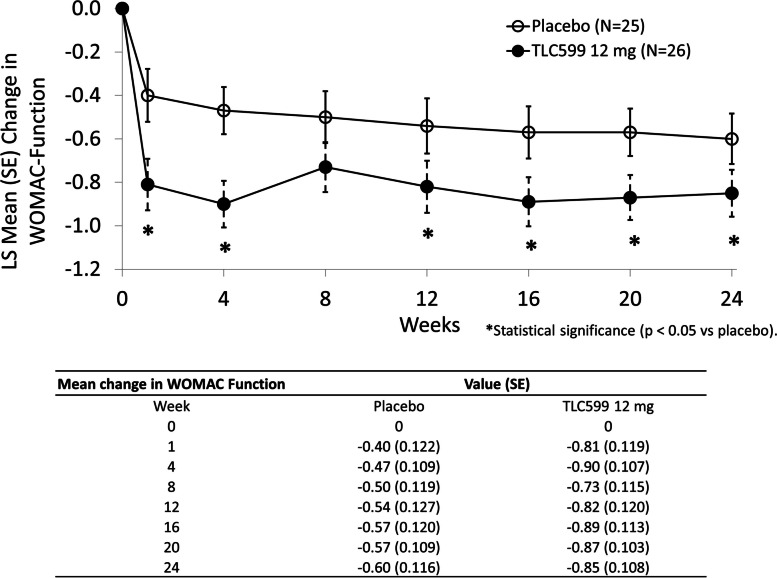
The mean change in WOMAC-Function scores through week 24. *p < 0.05. LS, least squares; SE, standard error

Improvement in physical function with TLC599 was assessed using the WOMAC-Function subscale. Participants receiving one injection of TLC599 12 mg displayed statistically significantly greater improvement in function than those treated with placebo at all time points except at week 8 (Fig. 4). TLC599 18 mg did not demonstrate significantly greater improvement in function in comparison with placebo at any time point.

**Fig. 5:**
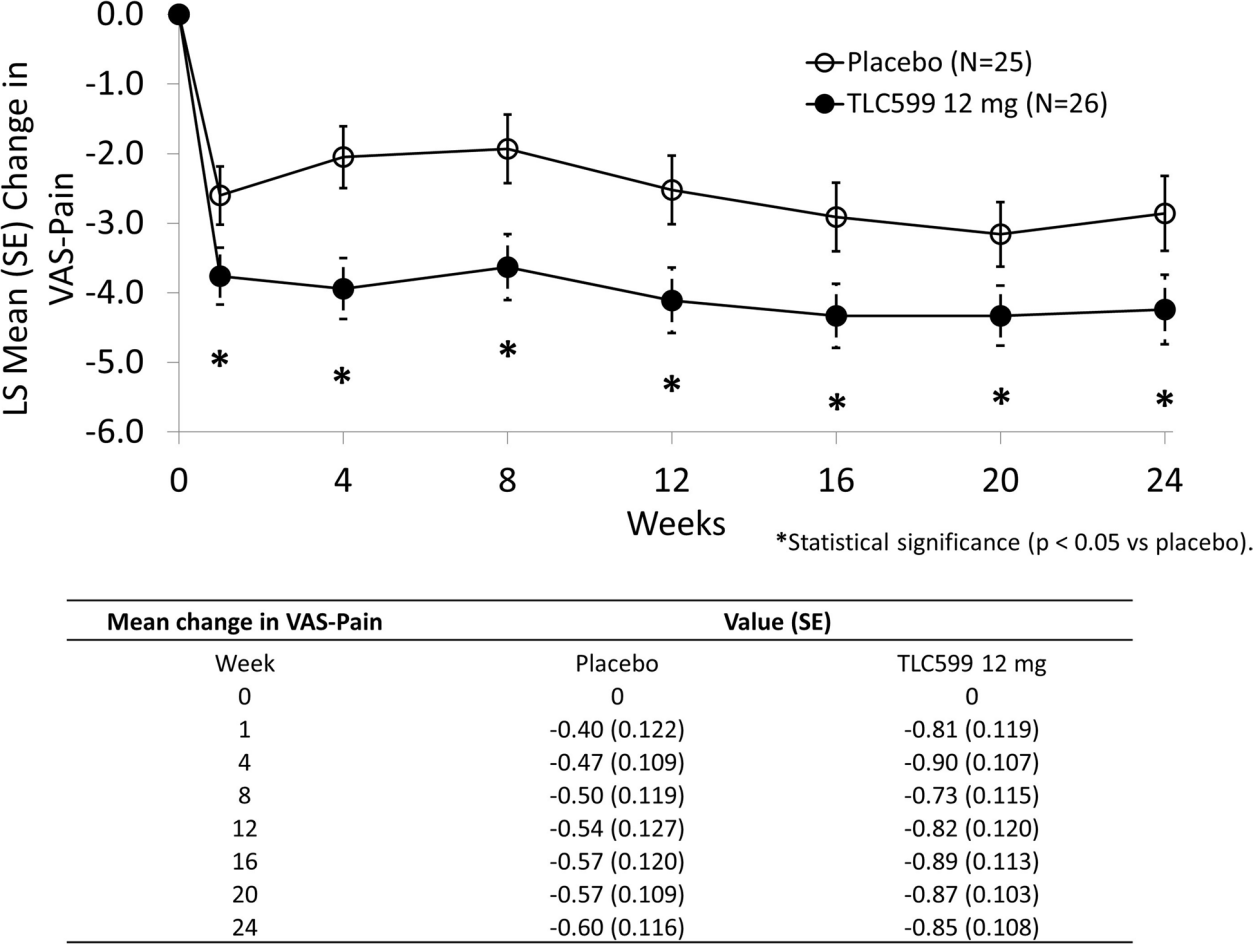
The mean change in VAS-Pain score. *p < 0.05. LS, least squares; SE, standard error

In the interval analyses of reduction in pain through week 24 utilizing the VAS, results were similar to those of the WOMAC-Pain. Concordant with the WOMAC-Pain results at the individual scheduled visit, TLC599 12 mg-treated participants also demonstrated a significantly greater reduction in pain compared to placebo at all time points, including week 24 visit (LS mean difference = − 1.38, *p* = 0.0319) (Fig. 5). TLC599 18 mg only demonstrated a significantly greater reduction in VAS pain in comparison with placebo at week 8.

Regarding the life of quality assessment utilizing EQ-5D questionnaires, improvements with both TLC599 group treatment groups were not statistically superior to placebo at most time points (data not shown).

**Fig. 6:**
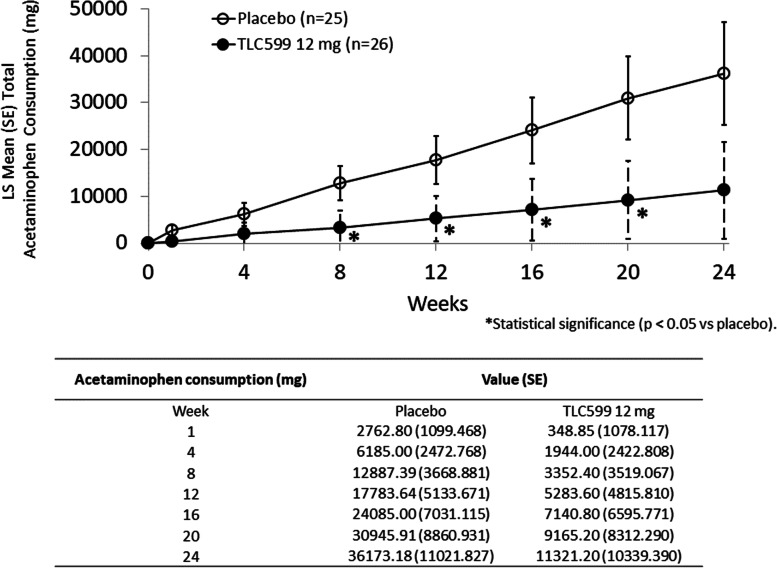
The mean acetaminophen consumption after single injection of study drug. *p < 0.05. LS, least squares; SE, standard error

The use of acetaminophen during the study was less than the placebo group in both of the TLC599-treated groups throughout 24 weeks. Acetaminophen use among the TLC599 12 mg-treated participants was significantly lower than that of the placebo group at most time points through week 20 (Fig. 6).

### Safety

Overall, 18 (69.2%) participants in the TLC599 12 mg group, 20 (83.3%) participants in the TLC599 18 mg group, and 17 (68.0%) participants in the placebo group reported at least 1 treatment-emergent AE (TEAE). Frequent adverse events are shown in Table 3. Treatment-emergent adverse events judged to be at least possibly related to the study treatment were reported in 26.9% of the participants treated with TLC599 12 mg and 45.8% of the participants treated with TLC599 18 mg (compared to 16% with placebo). Most TEAEs were mild or moderate in severity. There was 1 serious AE of clear cell renal cell carcinoma, reported for a participant in the TLC599 12 mg group, which was judged not related to study treatment. No deaths or TEAEs leading to participant discontinuation were reported in this study.

**Table 3:** Frequent (> 5%) treatment-emergent adverse events (safety population)

AE category	Placebo (***N*** = 25)	TLC599 12 mg (***N*** = 26)	TLC599 18 mg (***N*** = 24)
**Any adverse event**	**17 (68%)**	**18 (69%)**	**20 (83%)**
Headache	4 (16%)	3 (12%)	6 (25%)
Nasopharyngitis	3 (12%)	4 (15%)	1 (4%)
Cortisol decreased	0	2 (8%)	6 (25%)
Upper respiratory tract infection	2 (8%)	1 (4%)	4 (17%)
Arthralgia	3 (12%)	1 (4%)	2 (8%)
Glucocorticoid deficiency	0	3 (12%)	1 (4%)
Bronchitis	3 (12%)	0	0
Cough	0	0	2 (8%)
Diarrhea	0	3 (12%)	0
Dyspepsia	0	2 (8%)	0
Lipase increased	2 (8%)	0	1 (4%)
Toothache	1 (4%)	0	2 (8%)
Urinary tract infection	1 (4%)	2 (8%)	0
Chronic kidney disease	2 (8%)	0	0
Injury	2 (8%)	0	0

Laboratory abnormalities of blood cortisol levels at routine study visits were reported as TEAEs with coded terms of “cortisol decreased” in 8 participants and “glucocorticoid deficiency” in 4 participants (all in TLC599-treated subjects). All events were resolved, typically by the next scheduled laboratory assessment, and were not accompanied by associated signs and symptoms that might be attributed to hypocortisolism. No other notable laboratory trends were found in this study, and no deleterious effects on the study knees were observed by MRI in TLC599 and placebo treatment groups.

## Discussion

The modest duration of corticosteroids leads to a need for repeat injection for patients with chronic joint pain due to knee OA. However, the potential chondrotoxicity of common corticosteroid drugs such as triamcinolone acetonide also limits the frequency of its injection and restricts its clinical benefit for long-term pain management [12]. The development of the water-soluble corticosteroid dexamethasone sodium phosphate with both immediate-release and sustained-release profiles in the liposomal formulation is anticipated to solve this problem by providing satisfactory and durable pain relief and reducing the injection frequency. Notably, in toxicology studies in dogs and rabbits, neither cartilage damage nor proteoglycan loss was observed after repeated dosing with TLC599 [14].

The results from this clinical study have demonstrated TLC599’s long-acting efficacy in comparison with placebo. In contrast to another approved steroid product of sustained-release formulation [15], the current study has shown the duration of pain control of a single TLC599 injection could be up to week 24 without a decline in effect. A larger and well-designed pivotal study (ClinicalTrials.gov identifier: NCT04123561) is currently ongoing to confirm this efficacy profile. It was reported that reductions in chronic pain intensity in individuals of at least 30% appear to reflect at least moderate clinically important differences, and it is recommended that the percentages of patients responding with this degree of pain relief be reported in clinical trials of chronic pain treatments [16]. The dose level of TLC599 12 mg in this study demonstrated a defined durable reduction (> 30% reduction at each study visit) in pain and improvement in physical function.

Acetaminophen is a mild analgesic with little meaningful clinical benefit and real risks of harm, and the American Academy of Orthopedic Surgeons suggests no more than 3000 mg per day to minimize its risk of liver damage [17–19]. The current study allowed acetaminophen as the only rescue medication during the study period, and the TLC599 12 mg group was observed to consume significantly less acetaminophen than the placebo group, as assessed at most visits. TLC599 has the potential to reduce the need for oral medication use in the setting where opioids are frequently resorted to for pain control.

In contrast to the TLC599 12 mg dose, the 18 mg dose did not demonstrate statistically greater pain reduction and function improvement over placebo. The reasons for this are unknown and may be attributable to the small sample size in this study. However, this finding was consistent with an in-vitro drug release study of TLC599 at different dose levels (5.1, 8, 18, and 36 mg) in artificial synovial fluid, in which the rate of DSP release was greatly reduced at doses of 18 mg and higher (unpublished data), suggesting that doses less than 18 mg may be optimal for both immediate and sustained effect of the drug. As TLC599 12 mg showed greater efficacy compared to TLC599 18 mg, 12 mg can be considered the dose of choice for further clinical investigation of TLC599 in treating OA knee pain.

TLC599 was shown to be well tolerated in the current study. Although a reduction in cortisol was observed in a portion of participants at the beginning of the study (less in the TLC599 12 mg group than in the 18 mg group), such transient cortisol reduction is a well-described physiologic response after IA cortisol injections [20]. The pharmacodynamic reduction in cortisol typically evidenced recovery after 1 week and was not associated with any adverse signs or symptoms. The safety profile seen in participants who received TLC599 was generally consistent with that expected in an older population of OA patients receiving IA corticosteroid injections.

## Conclusions

The results of the current study suggest that TLC599 provides durable pain relief and functional improvement while maintaining a satisfactory safety profile for a time period well beyond that of current treatment options. Replication in a larger phase III study with adequate ethnic diversity will be required to confirm the reported findings.

## Data Availability

The datasets generated and/or analyzed during the current study are not publicly available due to commercial confidentiality reasons but are available from the corresponding author on reasonable request.

## Abbreviations

CI: Confidence interval
DSP: Dexamethasone sodium phosphate
ECG: Electrocardiogram
EQ-5D: EuroQol-5 Dimension
HbA1c: Hemoglobin A1c
IEC: Independent ethics committee
IRB: Institutional review board
IWRS: Interactive Web Response System
KL grade: Kellgren-Lawrence grade
LS: Least squares
mITT: Modified intent-to-treat
MMRM: Mixed-effects model for repeated measures
MRI: Magnetic resonance imaging
NSAID: Non-steroidal anti-inflammatory drugs
OA: Osteoarthritis
OARSI: Osteoarthritis Research Society International
PL: Phospholipid
SAS: Statistical Analysis System
SE: Standard error
TEAE: Treatment-emergent adverse event
VAS: Visual analog scale
WOMAC: Western Ontario and McMaster Universities Arthritis Index
